# Alterations of oral microbiota are associated with the development and severity of acute pancreatitis

**DOI:** 10.1080/20002297.2023.2264619

**Published:** 2023-10-05

**Authors:** Yiting Liu, Hang Liu, Yuping Rong, Qiao Shi, Qiang Yang, Hanjun Li, Zhengle Zhang, Jing Tao

**Affiliations:** a Department of Pancreatic Surgery, Renmin Hospital of Wuhan University, Wuhan, China; b Department of Clinical Laboratory, Institute of Translational Medicine, Renmin Hospital of Wuhan University, Wuhan, China

**Keywords:** Acute pancreatitis, oral microbiota, dysbiosis, diversity, prediction, 16S rRNA gene

## Abstract

Acute pancreatitis (AP) is a common abdomen clinical emergency. Most APs have mild clinical symptoms and a good prognosis. However, about 20% of patients develop severe acute pancreatitis (SAP), increasing morbidity and mortality. The microbiome’s impact on AP pathophysiology has received increasing attention. Hence, to explore changes in oral microbial composition in acute pancreatitis, we collected clinical information and oral saliva samples from 136 adult participants: 47 healthy controls, 43 acute mild AP (MAP), 29 moderate AP (MSAP), and 17 severe AP (SAP). Using 16S rRNA gene sequencing, 663,175 high-quality sequences were identified. The relative abundance and diversity of oral microorganisms in AP patients increased, with decreased beneficial bacteria such as *Streptococcus*, *Neisseria*, and *Gemella*, and increased *Prevotella, Veillonella, Granulicatella, Actinomyces*, and *Peptostreptococcus* in the AP group. Further changes in microbial composition occurred with increasing disease severity, including a decreased abundance of beneficial bacteria such as *Neisseria, Haemophilus*, and *Gemella* in MSAP and SAP compared to MAP. Moreover, the Lefse analysis showed that *Prevotella, Peptostreptococcus, Actinomyces*, and *Porphyromonas* were better microbial markers for AP. Therefore, oral microbiome changes could distinguish AP from healthy individuals and serve as an early novel predictor of disease severity in AP patients.

## Introduction

Acute pancreatitis (AP) is a common acute abdomen clinical emergency, usually due to abnormal activation of pancreatic enzymes caused by multiple etiologies, causing autodigestion of the pancreas [1]. The revised Atlanta criteria divide AP according to disease severity: mild acute pancreatitis (MAP), mild severe acute pancreatitis (MSAP), and severe acute pancreatitis (SAP) [2]. Most AP patients have mild clinical symptoms and a good prognosis. However, about 15–20% of patients develop SAP with systemic multi-organ failure, which is dangerous and has a high mortality rate [3]. Although many clinical serological indicators, scoring systems, and imaging findings have been developed to diagnose AP severity, the specificity and sensitivity of these biomarkers are still insufficient, or at the time of SAP diagnosis, the disease has progressed to a severe stage [4,5], lacking effective early warning and intervention indicators. Therefore, more reliable and accurate biomarkers are urgently needed for early disease diagnosis and assessment.

As the second largest microbiota besides the intestinal tract, the oral microbial community affects the body’s immune function, carcinogen metabolism and nutrient digestion [6]. It plays an important role in development of oral and pancreatic cancer, periodontal disease, cardiovascular disease, and rheumatoid arthritis [7]. Michaud D S et al [8] were the first to suggest an association of oral and fecal microbial composition with pancreatic ductal adenocarcinoma. Subsequently, Fan et al. [9] found that oral pathogenic microorganisms can be used as microbial biomarkers to predict pancreatic cancer occurrence. Several hypotheses exist to explain how oral microorganisms affect pancreatic inflammation and canceration. First, due to the anatomical factor that the pancreatic duct opens at the large duodenal papilla, oral microbiota can be ingested and colonize the esophagus, stomach, intestine, and bile duct, directly affecting the microbiota of the pancreas through reflux back to the pancreatic duct [10–12]. In addition, it can also communicate with digestive system organs through blood circulation and biliary transmission [13]. Diehl G E et al [14] demonstrate that the microbiota also indirectly acts on the gut via mesenteric lymph nodes in mice model. Beger H G et al [15] found that S*taphylococcus, Enterococcus*, and *Escherichia coli* could be cultured in necrotic pancreatic tissue in an early prospective clinical study, further support for this idea. Mechanically, Pushalkar S et al. [11] found *Bifidobacterium pseudolongum* in the pancreas of mice by oral gavage, and that this strain promotes tumorigenesis by differentially activating selective Toll-like receptors in monocytes to generate a tolerogenic immune program. In addition, *Porphyromonas gingivalis* can affect pancreatic disease by mediating toll-like receptor (TLR)-2/4, an innate immune and pro-inflammatory signaling molecule [16].

Thus, oral microbiota may be closely associated with pancreatic inflammatory diseases. However, few studies have focused on the correlation of oral microbiota with pancreatic inflammatory diseases. Saliva samples are convenient and easy to collect, which can reduce the economic and time burden of patients and improve the efficiency of sampling and analysis. On the other hand, saliva samples have higher stability than fecal samples [17]. In this study, we selected oral samples to explored the relationship between oral microbial alterations and the course and severity of AP patients.

## Materials and methods

### Study subjects

We collected saliva samples from a cross-sectional cohort of 136 subjects comprising 89 AP patients and 47 HCs for 16s rRNA gene sequencing. Patients were recruited between February 2022 and November 2022 at the Department of Pancreatic Surgery, Renmin Hospital of Wuhan University. Patients with AP should be enrolled within 72 hours of onset of symptoms and meet the following diagnostic criteria: (1) abdominal pain symptoms consistent with AP; (2) serum amylase or lipase activity at least three times higher than normal; (3) abdominal imaging changes consistent with AP [18]. Then, all the AP patients according to the disease severity were divided into three groups: MAP, MSAP, and SAP, based on the revised Atlanta criteria [2]. Subjects were excluded if they were under 18, pregnant, had oral-related diseases within the last month, taking antibiotics or intraprobiotic drugs, imaging suggestive of chronic pancreatitis and metabolic, liver, immunosuppressive diseases, and cancer. These HCs were healthy volunteers (medical examiners) from Renmin Hospital of Wuhan University. Their age and gender did not differ significantly from the patient population. The HC group must meet the following inclusion criteria: free of metabolic, cardiovascular, intestinal or oral-related diseases; no pregnancy; no antibiotic or probiotic medications in the last 3 months. The investigation conformed with the principles outlined in the Declaration of Helsinki. Informed consent was obtained from all subjects before enrollment. This study was approved by the Ethics Committee of Renmin Hospital of Wuhan University (WDRY2019-K068).

### Sample collection

All subjects were asked to avoid brushing, eating, or chewing gum for 12 h before sample collection. Subjects were asked to gargle with 10 mL of saline for one minute and spit the gargle into a 50 mL sterile centrifuge tube. Samples were transferred to the laboratory for storage −80°C within two hours.

### 16S rRNA gene sequencing

According to the E.Z.N.A.® soil DNA kit (Omega Bio-tek, Norcross, GA, U.S.) instructions, the total DNA of the microbial community was extracted, and the DNA concentration and purity were determined by NanoDrop2000, PCR amplification on the V3-V4 variable region of the 16S rRNA gene was conducted Using the following primers: 338F (5’-ACTCCTACGGGAGGCAGCAG-3'); 806 R (5’-GGACTACHVGGGTWTCTAAT-3'). The PCR amplification of the 16S rRNA gene was performed as follows: initial denaturation at 95°C for 3 min, followed by 27 cycles of denaturing at 95°C for 30 s, annealing at 55°C for 30 s, and extension at 72°C for 45 s, and single extension at 72°C for 10 min, and end at 4°C. The PCR mixtures include 5× TransStart FastPfu buffer, 4 μL; 2.5 mM dNTPs, 2 μL; 5 μM forward primer, 0.8 μL; 5 μM reverse primer, 0.8 μL; TransStart FastPfu DNA Polymerase, 0.4 μL; template DNA, 10 ng; and ddH2O up to 20 μL. Purified amplicons were pooled in equimolar and paired-end sequenced on an MiSeq PE300 platform (Illumina, San Diego, USA) according to the standard protocols by Majorbio Bio-Pharm Technology Co. Ltd. (Shanghai, China). The raw reads were deposited into the NCBI Sequence Read Archive database (BioProject number: PRJNA935469).

### Bioinformatics and statistical analysis

Raw 16S rRNA gene sequencing reads were demultiplexed, quality-filtered by fastp (https://github.com/OpenGene/fastp,version 0.20.0), and merged using FLASH(http://www.cbcb.umd.edu/software/flash, v.1.2.7). The Fastp and FLASH parameters used for quality filtering and merging are as follows: 1) Filter reads with mass values below 20 at the end of the reads, set a window of 50bp, if the average mass value within the window is below 20, truncate the back-end subtracted bases starting from the window, filter the reads with quality control below 50 bp, and remove the reads containing N bases. 2) Based on the overlap relationship between PE reads, splice (merge) pairs of reads into a sequence with a minimum overlap length of 10 bp. 3) The maximum mismatch ratio allowed in the overlap region of the spliced sequence is 0.2 to screen for non-conforming sequences. 4) Distinguish the samples according to the barcode and primers at the beginning and end of the sequence, and adjust the sequence orientation, the allowed number of mismatches for barcode is 0, and the maximum number of primer mismatches is 2.

The OTUs with 97% similarity cutoff were clustered using UPARSE 7.1, and chimeric sequences were identified and removed. The taxonomy of each OTU representative sequence was analyzed by RDP Classifier 2.2 against the 16S rRNA database (e.g, Silva v138) using a confidence threshold of 0.7. Alpha diversity indices (Shannon and Simpson index) were performed using the vegan package, and the beta diversity was conducted by adopting the principal-coordinate analysis (PCoA; Bray-Curtis algorithm). Bacterial taxonomic analyses and comparisons including bacterial phylum and genus were conducted between two groups using the Wilcoxon rank sum test. Based on the normalized relative abundance matrix (Data analysis using relative abundance of species in each sample), features with significantly different abundances between assigned taxa were determined by Linear discriminant analysis Effect Size (LEfSe) with the Kruskal-Wallis rank sum test (*p* < s0.05), LDA was used to assess the effect size of each feature (cutoff LDA score ≥ 3.5). Random forest models were constructed using the R package (v. 4.6–14), and the ROC curves were plotted by the ‘pROC’ package (v. 1.17.0). PICRUSt predicted KEGG pathways functions based on 16S rRNA sequencing data. The predicted KEGG results were compared between groups according to the sequence number using the Wilcoxon rank-sum test. A *p* value < 0.05 were considered statistically significant. If the data were normally distributed and did not violate homogeneity, it was evaluated by the Student’s t-test, and one-way analysis of variance (ANOVA) for more than two groups. Non-normally distributed variables were compared with the Wilcoxon rank test (two groups).

## Results

### 16S rRNA gene sequencing characterization of oral microbes in AP patients

**Figure 1.**
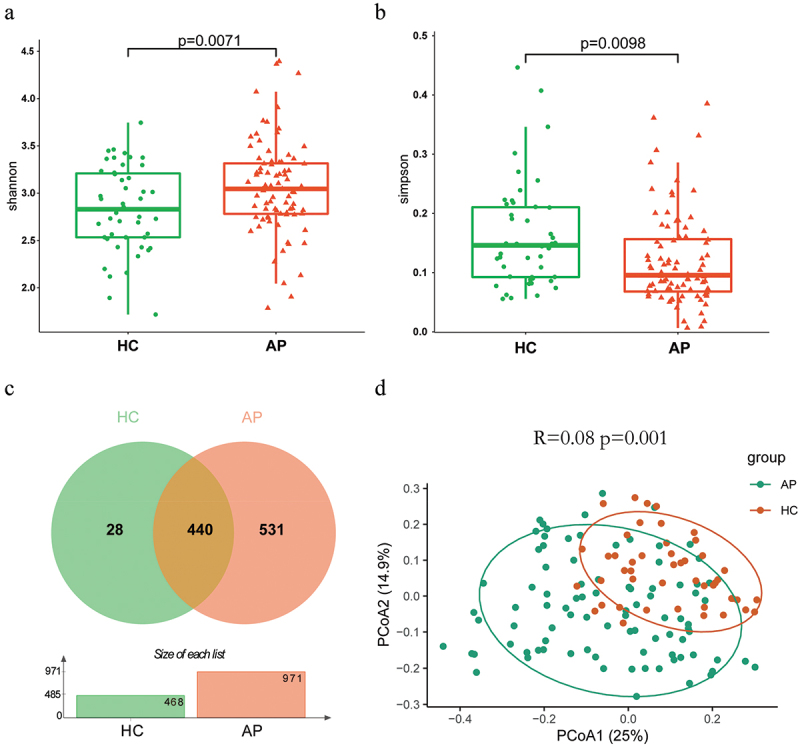
Alpha and beta diversity analysis between AP and HC groups. (a) Alpha diversity based on the Shannon index; box-plot features represent the median (central line), upper and lower quartiles (box), and the maximum and minimum values of the data (bars). (b) Alpha diversity based on the Simpson index. (c) venn diagram reflecting the similarity and repeatability of species composition between two groups. (d) beta diversity based on the PCoA plot. Each symbol represents the gut microbiota of a sample. AP, acute pancreatitis; HC, healthy control.

We included 136 eligible cases: 47 healthy controls (HCs), and 89 AP (43 MAP, 29 MSAP, and 17 SAP). Then, we performed 16S rRNA gene sequencing to explore the differences in oral microbiota between AP patients and healthy controls. A total of 6,631,275 high-quality sequences with an average length of 424 bp were identified, resulting in 1032 OTUs (operational taxonomic units), 29 phylum, 70 classes, 161 orders, 255 families, 472 genus, and 819 species (Table S1). The species accumulation curves for all samples reached a horizontal state after a very high rise, indicating the adequacy of our sample collection efforts (Figure S1). Alpha diversity indices were calculated to assess the differences in colony diversity between the two groups. According to the Shannon (Figure 1a), Simpson (Figure 1b), Heip (Figure S1B), Chao (Figure S1C) and Sobs (Figure S1D) indexes, the bacterial community richness of the AP group was significantly higher than the HC group (Table S2). Additionally, 531 of the 971 OTUs were shared by HC and AP groups, and 440 were unique to the AP group. The histogram showed that the AP group had more OTUs than the HC group, consistent with the Alpha diversity results (Figure 1c). The Bray-Curtis principal coordinate analysis (PCoA) for the microbiome space between samples showed that the microbial composition of the HC and AP groups was significantly separated (*R* = 0.1558, *p* = 0.001) (Figure 1d).

### Taxonomic alterations of oral microbiota in AP patients

**Figure 2.**
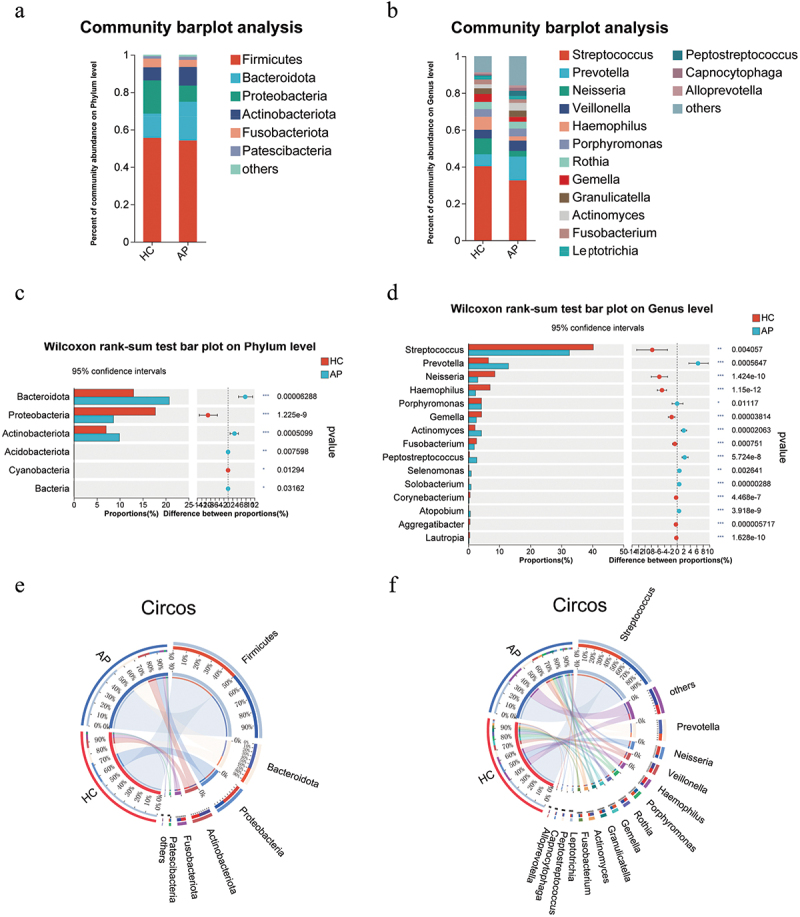
Oral microbiota composition. average bacterial community composition at the phylum (a) and genus levels (b) levels. Analysis of species differences in oral flora between AP HC groups at the phylum(c and genus (d) levels. On the left, the X- and Y-axis represents the average relative abundance of the gut microbiota species in various groups and the gut microbiota species names at a certain classification level, respectively. On the right, the X- and Y-axis represents different gut microbiota species between various groups and *p* value of significance. *0.01 < *p* < 0.05, **0.01 < *p* < 0.001, ***p < 0.001. Circos diagram of microbial composition at the phylum(E) and genus(F) level in the three groups (left and right semicircles represent different samples and different species).

The bacterial richness analysis revealed significant differences in the oral microbial composition between HC and AP groups (Figure 2). The top five oral microorganisms in the HC and AP groups at the phylum level were *Firmicutes, Bacteroidota, Proteobacteria, Actinobacteriota*, and *Fusobacteriota*. *Bacteroidota* and *Actinobacteriota* were enriched in AP compared to HC saliva, while *Proteobacteria* were reduced (Figure 2a). At the genus level, 15 genera, such as *Streptococcus, Prevotella, Neisseria, Veillonella, Haemophilus, Porphyromonas, Rothia*, and *Gemella* accounted for an average of more than 90% in both groups (Figure 2b). *Streptococcus, Neisseria, Haemophilus*, and *Gemella* were reduced in the AP group, whereas *Prevotella, Veillonella, Granulicatella, Actinomyces*, and *Peptostreptococcus* increased compared to the HC group. We then used the Wilcoxon rank sum test to analyze the significant differences in microbial composition between the two groups. *Bacteroidota, Actinobacteriota* and *Acidobacteriota* were significantly higher in the AP group than in the HP group at the phylum level (Figure 2c). On the other hand, *Proteobacteria* and *Cyanobacteria* were significantly reduced in the AP group. *Streptococcus, Neisseria, Haemophilus, Gemella, Fusobacterium, Corynebacterium*, and 29 other genera were significantly reduced (*p* < 0.05) compared to the HC group (Figure 2b). The analysis at the phylum and genus level showed significant differences in species composition between the two groups (Table S3 and S4). Differences in class, family, and species levels were also separately detected (Figure S2). Additionally, we constructed Circos plots to visualize the microbial composition between the two groups at the phylum (Figure 2e) and genus level (Figure 2f), and the results were consistent with the description above.

### Potential AP-associated oral microbial biomarkers

**Figure 3.**
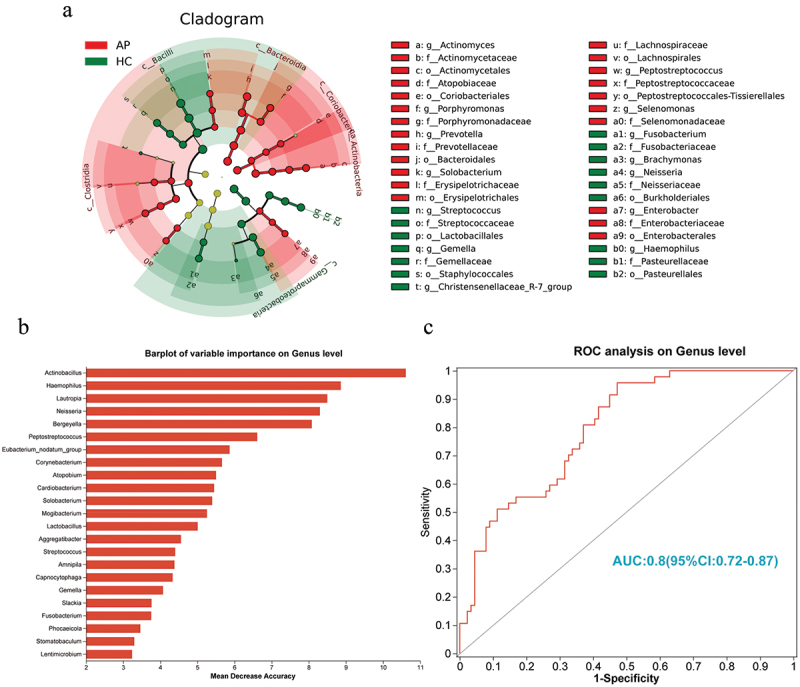
LEfSe and LDA based on OTUs among AP and HC groups. (a) the circles radiating from the inside to the outside represent the classification level from the phylum to the genus. Each circle of a level represents that at level’s classification, and the circle’s diameter represents its relative abundance. Biomarkers with significant differences are colored according to the grouping color (‘p’ represents phylum, ‘c’ represents class, ‘o’ represents order, ‘f’ represents family, ‘g’ represents genus). (b) the top 23 biomarker bacterial classes were identified by applying random forests regression to their relative abundance values. (c) we constructed receiver operating characteristic curves (ROCs) for optimized microbial biomarkers using the top 23 microbial markers identified by random forest regression to better distinguish between the HC and AP groups. AUC: area under the ROC curve.

Furthermore, we performed LefSE analysis to distinguish the bacterial abundance differences that might be associated with AP disease progression (Figure 3). First, we analyzed the taxonomic units of the oral bacterial flora between the HC and AP groups (Figure 3a) to show the phylogenetic distribution of the oral microbiota between the two groups. We restricted the LDA score to > 3.5 to detect valid biomarkers (Figure S3). The histogram showed the microorganisms with significant differences between the two groups. Seven genera dominated AP: *Prevotella, Peptostreptococcus, Actinomyces, Enterobacter, Selenomonas, Solobacterium*, and *Porphyromonas*. In contrast, *Streptococcus, Neisseria, Gemella, Brachymonas*, and *Fusobacterium* were better biomarkers for HC microbiota (Table S5). Then, we constructed a random forest model with 10-fold cross-validation between the two groups and found potential biological markers that could distinguish between HC and AP patients, similar to the LefSE analysis results (Figure 3b). Using the top 23 species of importance, we construct a receiver operating characteristic (ROC) curve of optimized microbial biomarkers to distinguish better HC and AP groups (AUC: 0.80; 95% CI: 0.72–0.87). These results showed that the selected oral microbial markers were accurate for HC and AP groups (Figure 3c).

### Taxonomic characterization of the oral microbial profile across AP severity

**Figure 4.**
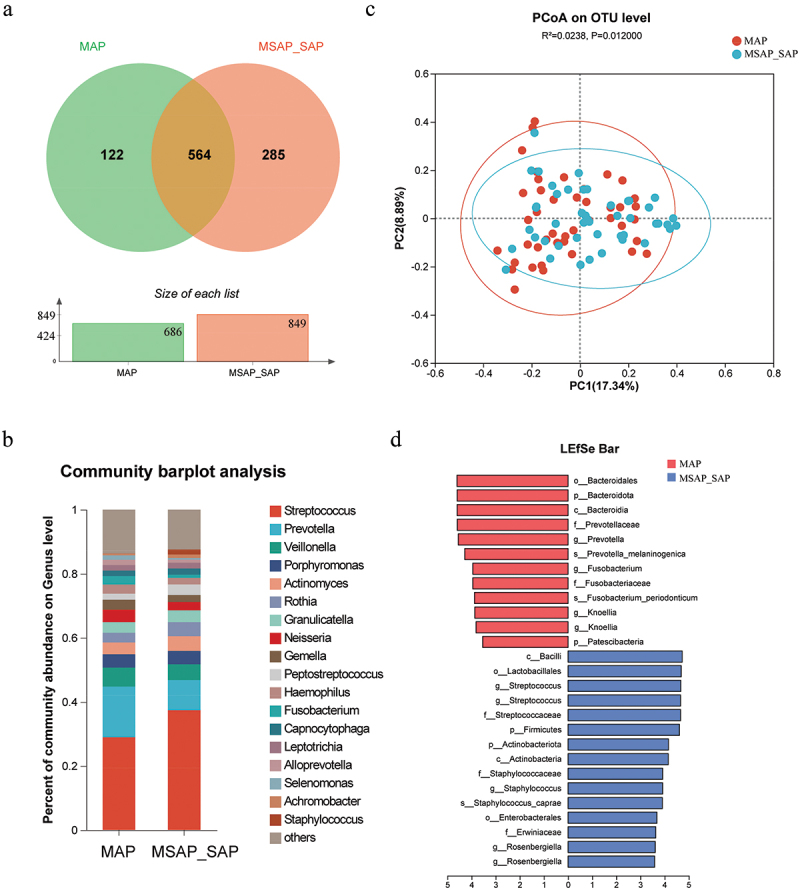
Differences in oral microbiota between MAP and MSAP_SAP groups. (a) venn diagram reflecting the similarity and repeatability of species composition between two groups. (b) average composition of bacterial community at the genus levels. (c) beta diversity analysis based on the PCoA plot. (d) microbes with differential abundance (LDA score＞3.5) were plotted as a histogram. LDA score histogram showing the oral microbiome with significant differences between the two groups.

Furthermore, we characterized oral microbiota changes during AP progression. MSAP and SAP have more serious clinical manifestations and poorer prognoses than MAP. Thus, we combined MSAP and SAP into one group and analyzed it compared to MAP. The Venn diagram (Figure 4a) indicated that both groups shared 564 OTUs, and 285 were unique to MSAP and SAP. The histogram showed that the total number of OTUs in the MSAP and SAP groups increased compared to the MAP group. The differences in oral microbiota communities at the genus level between the two groups (Figure 4b) were as follows: *Neisseria, Haemophilus*, and *Gemella* decreased, and *Actinomyces, Peptostreptococcus*, and *Atopobium* increased in MSAP and SAP. The PCoA plot (Figure 4c) showed that MSAP and SAP had a distinct microbial, distribution from the MAP group (*R* = 0.0238, *p* = 0.012). The oral bacteria composition differences at the phylum and species levels were separately presented in Figure S3. The LEfSe analysis revealed a significant increase of *Staphylococcus* and *Rosenbergiella* and a substantial reduction of *Prevotella* and *Fusobacterium* in MSAP and SAP compared to MAP (Figure 4d).

### Altered microbial functions in AP

**Figure 5.**
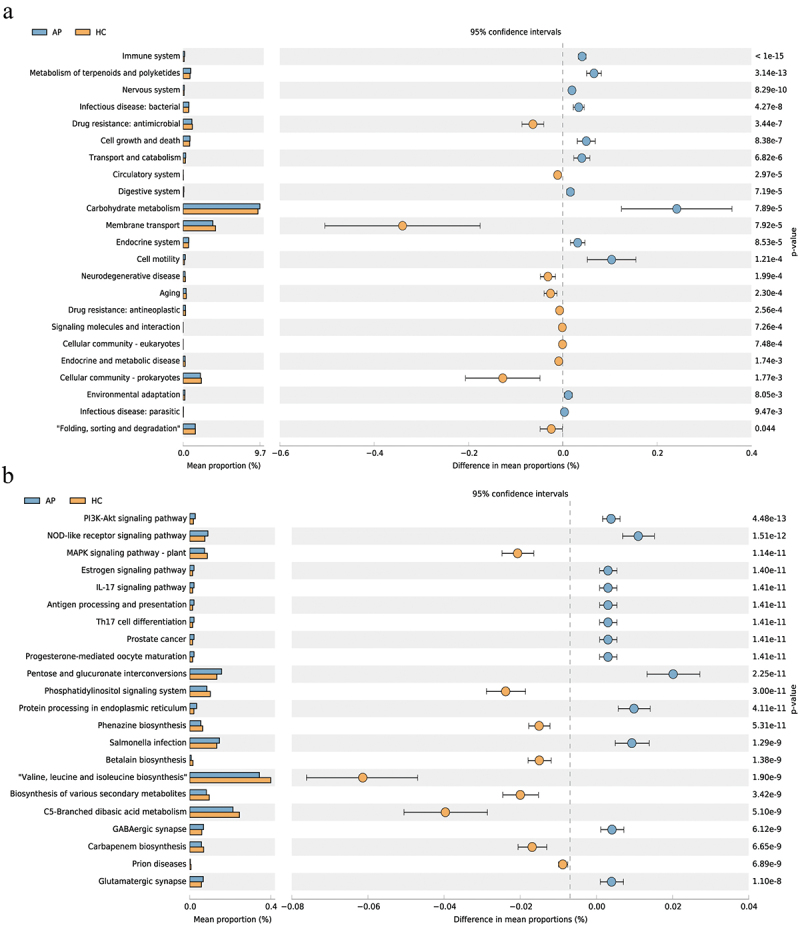
Oral microbial functional dysbiosis in AP patients and HCs. differential KEGG pathways by PICRUSt. Significant differences between AP and HC groups at level 2 (a) and level 3 (b).

To study the functional and metabolic changes in the oral microbial community of AP patients, we predicted the functional composition of the microbial community using the Phylogenetic Investigation of Communities by Reconstruction of Unobserved States (PICRUSt) (Figure 5). Differences in metabolic pathways between the two groups were distinguished using the Wilcoxon rank sum test. 42 KEGG pathways were predicted, and 23 significantly differed between the two groups based on the number of sequences correlated with the pathways. 12 pathways were significantly enhanced in the AP group: ‘Infectious disease: bacterial’, ‘Digestive system’, ‘Nervous system’, ‘immune system’, ‘Cell motility’, ‘Cell growth and death’, and ‘Carbohydrate metabolism’ (Figure 5a). In contrast, the oral microbiome of the HC group was characterized by ‘Drug resistance: antineoplastic’, ‘Cellular community-prokaryotes’, and ‘membrane transport’. We detected a significant increase in the bacterial invasion of epithelial cells in the bacterial infectious disease pathway for the AP group (Figure 5b). Hence, the PICRUSt2 analysis demonstrated the dysfunctional microbial composition of the two groups and the metabolic dysfunction of the microbial community.

### Clinical factor data from different groups related to oral microbiota

**Figure 6.**
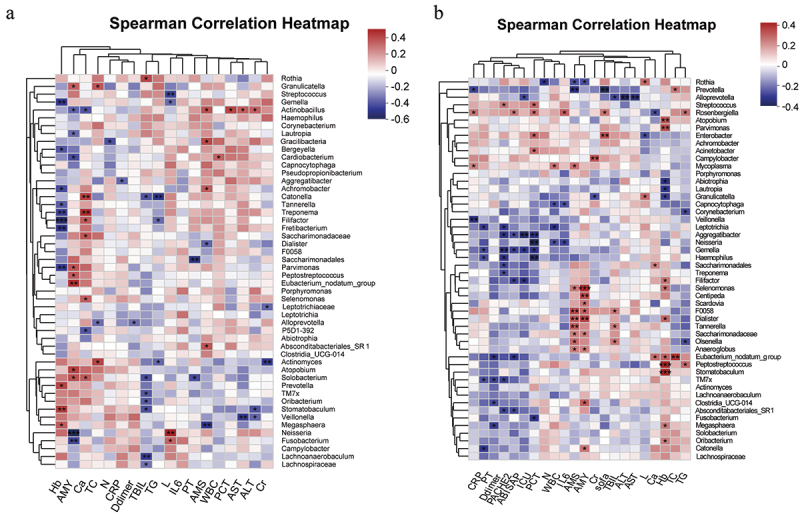
Associations between oral microbiomes and clinical indices of AP. Spearman correlations between the top 50 species regarding total taxonomic abundance, clinical outcomes, and disease severity indicators. Positive (red) or negative (blue) correlations are shown by a two-color heatmap, with asterisks denoting statistical significance (*p < 0.05, **p < 0.01).

The HC and AP groups did not differ regarding age, gender, ALT, and PT (Table 1). Meanwhile, serum inflammatory markers CRP (*p* < 0.0001), PCT (*p* < 0.0001) and IL-6 (*p* < 0.0001) were significantly higher in the MSAP and SAP groups, comprising a more severe disease compared to the MAP group. Moreover, we found that Ca^2+^ was significantly higher, and D-dimer was significantly lower in the MSAP and SAP groups. We used Spearman’s rank test analysis to investigate the correlation of the relative abundance of the top 50 most abundant genera with clinical indicators and disease severity (Figure 6). In the HC group, Hb was positively correlated with Gemella (r = -0.4, *p* = 0.005), Tannerella (r = -0.33, *p* = 0.02), and Filifactor (r = -0.47, *p* = 0.0007), while negatively correlated with Prevotella and Stomatobaculum (Figure 6A). The dominant genus *Rosenbergiella* in the AP group was positively correlated with CRP (*r* = 0.23, *p* = 0.02), IL-6 (*r* = 0.27, *p* = 0.01), PCT (*r* = 0.24, *p* = 0.02), BISAP score (*r* = 0.22, *p* = 0.03) and Sofa score (*r* = 0.23, *p* = 0.02). *Enterobacter* was positively correlated with Sofa (*r* = 0.29, *p* = 0.006) and PCT (*r* = 0.26, *p* = 0.001). Significantly reduced beneficial *Neisseria* in the AP group were negatively correlated with IL-6 (*r* = −0.23, *p* = 0.02), WBC (*r* = −0.21, *p* = 0.04), and PCT (*r* = −0.33, *p* = 0.001). Similar results (negative correlation with inflammatory indicators) were found for other beneficial bacteria significantly reduced in AP patients. For example, *Gemella* was negatively correlated with PCT (*r* = −0.26, *p* = 0.02), APACHE2 score (*r* = −0.28, *p* = 0.007), BISAP score (*r* = −0.31, *p* = 0.02) and ICU days (*r* = −0.25, *p* = 0.01); *Haemophilus* was negatively correlated with PCT (*r* = −0.23, *p* = 0.003), APACHE2 (*r* = −0.27, *p* = 0.001) and PT (*r* = −0.21, *p* = 0.04) (Figure 6B).

**Table 1.** Demographic characteristics of participants.

	AP			HC	
	MAP(*n* = 43)	MSAP_SAP (n = 46)	*p* value^1^	n = 47	*p* value^2^
Age(years)	44.7 ± 12.7	49.7 ± 16.6	0.025	43.7 ± 9.4	0.059
Sex (male/female)	20/23	22/24	0.901	22/25	0.966
BMI (kg/m2) Smoke, n (%) Drink, n (%) Hypertension, n (%) Diabetes, n (%)	24.6 ± 3.6 9 (20%) 6 (13.9%) 12 (29.7%) 13 (30%)	25.8 ± 5.9 12 (26%) 11 (23.9%) 14 (30%) 16 (34.7%)	0.578 0.567 0.541 0.793 0.647	23.8 ± 2.62 9 (19%) 8 (17%) 10 (21.2%) 8 (17%)	0.026 0.552 0.547 0.318 0.052
AMY (u/L)	283 (150–744)	547 (238–930)	0.055	69 (58–80)	<0.0001
LPS (u/L)	1746 (413–4000)	2022 (790–5345)	0.25	153 (95.7–195)	<0.0001
WBC (10^9^/L)	10.8 ± 4.2	13 ± 4.5	0.941	6.3 ± 1.6	<0.0001
Neu (10^9^/L)	75.5 ± 11.4	84.1 ± 6.5	<0.0001	60.3 ± 6.2	<0.0001
Hb (g/L)	140.3 ± 21.5	133.1 ± 36	0.006	152.8 ± 8.5	<0.0001
Lym (10^9^/L)	13.5 (9–21.7)	8.2 (5.8–11.4)	<0.0001	37.4 (32.4–41.4)	<0.0001
CRP (mg/dL)	31.8 (9.8–77.1)	104 (47–162)	<0.0001	1.6 (0.27–2.35)	<0.0001
PCT (ng/mL)	0.09 (0.04–0.2)	0.79 (0.35–2.58)	<0.0001	0.05 (0.02–0.08)	<0.0001
IL-6 (Pg/ml)	33.1 (20–55)	77.8 (30.2–278)	<0.0001	4.2 (2.3–6.5)	<0.0001
ALT (U/L)	26 (19–48)	36 (16–118)	0.307	31 (20–41.2)	0.228
AST (U/L)	24 (16–38)	39 (27–89)	<0.0001	26 (19.7–33)	0.041
Cr (μmol/L)	66 (53–74)	65 (50–104)	0.616	75.5 (63.7–87.2)	0.006
TBIL (mmol/L)	16.7 (10.6–25.2)	23 (14–41.2)	0.017	14.4 (7–20)	<0.0001
TC (mmol/L)	3.9 (3.4–5.1)	3.6 (3–5.8)	0.359	3.2 (1.9–4.5)	0.001
TG (mmol/L)	2 (0.8–5.7)	1.6 (0.8–10)	0.920	1 (0.5–1.5)	<0.0001
Ca^2+^ (mmol/L)	2.15 (2–2.3)	2 (1.8–2.3)	0.011	2.3 (2.2–2.4)	<0.0001
D-dimer (mg/L)	0.4 (0.2–0.8)	1.7 (0.4–3.7)	<0.0001	0.23 (0.07–0.34)	<0.0001
PT (sec) BISAP APACHE2 SOFA	11.6 ± 1.3 0 (0, 0) 1 (0, 3) 0 (0, 1)	12.7 ± 1.9 1 (0, 2.5) 5 (3, 9) 2 (1, 3.5)	0.032 < 0.0001 < 0.0001 < 0.0001	11.1 ± 1.1	0.57

Table 1 BMI: body mass index; AMY: Serum amylase; LPS: Serum lipase; WBC: white blood cell; Neu: neutrophil; Hb: Hemoglobin; Lym: lymphocyte; CRP: C-reaction protein; PCT: procalcitonin; IL-6: Interleukin-6; ALT: Alanine transaminase; AST: Aspartate Transaminase; Cr: creatinine; TBIL: Total bilirubin; TC: total cholesterol; TG: Triglycerides; PT: Prothrombin Tim; BISAP: Bedside Index for Severity in Acute Pancreatitis score; APACHE2: Acute Physiology and Chronic Health Evaluation II; SOFA: Sequential Organ Failure Assessment score. 1, HC Vs. AP 2, MAP Vs. MSAP_SAP.

## Discussion

As a habitat for many microorganisms, the oral cavity plays various of functions, such as forming an oral mucosal barrier, participating in immune responses, and resisting the invasion by foreign microorganisms. Periodontitis pathogens can stimulate cells to produce inflammatory factors such as IL-1β, IL-6, and TNF-α [19] and enter the body circulation system through the broken gingival epithelium in periodontal pockets, causing bacteremia, or ectopic colonization in other organs [20]. The oral microbiota is involved in the development and progression of inflammatory diseases such as inflammatory bowel disease [21] and cholangitis [22], and associated cancers such as colorectal tumors [23] and pancreatic cancer [24]. However, the correlation between oral microorganisms and AP has not been adequately studied, and this study will shed light on the correlation between AP and oral microbiota. This study was the first to describe the altered oral microbiota in AP patients from five aspects: species diversity, microbial community composition, diagnostic prediction model, functional prediction model, and correlation with clinical indicators.

We conducted a cohort study with 137 Chinese individuals, including 89 AP patients (43 MAP and 46 MSAP and 17SAP) and 48 HC using 16s rRNA gene sequencing, and found that AP was associated with oral salivary microbiota dysregulation. Regarding the oral microbial diversity between HC and AP groups, the Shannon index was higher in the AP group than in the HC group, suggesting AP group presented a higher oral microbial diversity, in consistent with the Simpson indices. Similar results were previously obtained on tongue samples from pancreatic cancer patients [25]. However, the relative gut microbiota abundance and diversity of AP patients is reduced compared to healthy individuals [26], different from our current findings regarding the oral microbiota. Existing studies have shown that oral microbes differ from intestinal ones, and increased diversity of oral microbiota often indicates inflammation and disease [27], Similarly, some harmful bacteria might increase and participate in disease occurrence and development [28,29]. Overall, we found significant changes in the oral microbiota composition in the AP group compared to the HC group, Thus, these changes might provide fundamental support for diagnosing and predicting of AP.

Moreover, we systematically described the differences in oral salivary microbiota composition between AP and HC. The oral microbial composition in MSAP and SAP significantly differed from MAP, suggesting that oral microbial composition might affect AP severity. *Streptococcus*, the most abundant beneficial bacterium at the genus level, decreased in the AP group, as well as the commensal bacteria *Neisseria* and *Gemella*. These results were similar to the study of oral microbiota in pancreatic cancer patients [30]. *Streptococcus*, an early colonizing bacterium of the oral cavity, reduces the inflammatory response by downregulating the nuclear transcription factor NF-кB in small intestinal epithelial cells, inhibiting PPARγ transcriptional activity and reducing IL-8 secretion, which contributes to immune regulation and host defense processes [31]. Furthermore, *S. salivarius K12* is now widely used as a probiotic to prevent and treat recurrent pharyngitis, tonsillitis, and pharyngitis [32,33]. It reduces the colonization of harmful bacteria in the oral and respiratory tracts by producing bacteriocin-like inhibitory substances (BLIS), promotes the balance of the organism’s microbiome, protects the host from inflammation and avoids apoptosis [33]. The oral symbiotic bacterium *Neisseria* promotes human health and participates in bioremediation, preventing oral diseases and reducing the risk of pancreatic cancer [24,34]. Gemella, a fibrin degrader, produces short-chain fatty acids (SCFAs) in the colon. SCFAs are important mediators of intestinal immune regulation and maintain intestinal barrier function by inhibiting the production of pro-inflammatory factors [35,36]. *Gemella* is reduced in AP patients, so the above response is inhibited, leading to intestinal bacteria displacement and causing AP or even aggravating. The decreased *Streptococcus* and increased *Prevotella* in AP are consistent with previous inflammatory bowel disease (IBD) studies [37].

Additionally, we suggested that some alterations in the AP oral microbiota could potentially increase their pathogenicity. The increase of opportunistic pathogenic bacteria, such as *Prevotella, Actinomyces* and *Veillonella*, might affect AP occurrence and development. For example, *Prevotella* predominates in periodontitis [38,39] and could exacerbate inflammatory disease by stimulating the production of inflammatory mediators such as CCL20, IL-8 and IL-6 [40]. Therefore, *Prevotella melaninogenica* is usually cultured as the sole infectious agent of pleuropneumonia, endocarditis, intra-abdominal infection, wound infection, necrotizing fasciitis, and purulent infection [41]. *Veillonellabe* members are associated with multiple infections and are significantly increased in alcoholic liver disease, primary biliary cholangitis (PBC), and other liver diseases, and Han M et al. [42,43] demonstrated the deleterious effects of *Veillonella* on PBC.

The Lefse results showed that beneficial commensal bacteria *Neisseria, Haemophilus*, and *Gemella* were reduced in MSAP and SAP and conditionally pathogenic bacteria such as *Prevotella* and *Fusobacterium* were increased compared to MAP. These results reinforce that a decrease in beneficial commensal bacteria and an increase in opportunistic anaerobes may have been involved in the development of AP and continue to impair the health of AP patients. Various oral and gut microbial models are available for the diagnosing and predicting cancer, such as oral [44], pancreatic [34], rectal [29], and liver cancer [28], and other diseases such as IBD [45], coronary heart disease [46], acute endocarditis [47], and Alzheimer’s disease [48]. Similarly, we believe the oral microbiome can be a biological marker to predict AP. Therefore, we used LefSE analysis to identify some oral microbial markers to separate AP patients from HCs, after constructing a random forest model to perform a 10-fold crossover to validate their diagnostic effect. *Selenomonas*, *Solobacterium*, and *Enterobacter* population are not suitable as microbial markers for identifying AP patients from HCs because of their low population levels. Therefore, *Prevotella, Peptostreptococcus, Actinomyces*, and *Porphyromonas* are better microbiota markers for AP.

Next, we performed a functional composition analysis of the microbial community between the two groups using PICRUSt. The membrane transport pathway was reduced in AP patients compared to HCs. In contrast, pathways associated with oxidative stress and toxicology increased in the AP group (e.g. terpenoids and polyketides and carbohydrate metabolisms). Furthermore, the cell motility pathway was excessive in the AP group. We know that as products of bacterial lysis, flagella can activate the epithelial expression of pro-inflammatory cytokines and chemokines [49] and that bacterial chemotaxis and motility can promote their colonization, exacerbating the inflammatory response [50]. The significant increase in the route of bacterial invasion into epithelial cells may indicate that potential oral pathogens adhere to and multiply on the surface of host cells, pass through the epithelial or endothelial host barrier, and enter the interior for activities [51]. Moreover, *Enterococci* and *Enterobacteriaceae* migrate from the gut to the circulation and pancreas, possibly leading to sepsis and pancreatic necrosis [52]. However, the basic pathways by which oral microbiota affect pancreatic diseases need further in-depth study.

The association between oral microbiota and clinical parameters provides new insights into the potential relationship between the oral microbiome and AP. We can determine AP progression using inflammation indicators such as WBC, PCT, IL-6, CRP, D-dimer, and Ca^2+^, and various scoring scales, such as APACHE2, BISAP, and Sofa score [53,54]. Decreased *Neisseria*, *Haemophilus*, and *Gemella* in AP were negatively correlated with clinical indicators responding to disease severity. The significant decrease in symbiotic *Neisseria* and *Gemella*, beneficial oral bacteria, affects AP development. These results demonstrated the close correlation between oral microbiota and AP severity.

This study has some limitations that need to be addressed. First, although the number of smokers did not differ between AP and healthy patients, the duration of smoking may change oral microbes, which may have an impact on the results [55]. Second, the 16s rRNA sequencing we used cannot provide more accurate species level and functional prediction, but we investigate the relationship between oral microbiota and AP, and the Metagenomics approach should be able to solve this problem. Finally, our article explores differences in oral microbiota between AP and healthy individuals, however, the pathways by which the oral microbiota affects pancreatic tissue and whether the inflammatory state of the oral microbiome in AP patients is a cause or a consequence of deterioration need to be further investigated at the molecular level, at both the cytological and zoological levels.

## Conclusion

In summary, we described the differences between the oral microbes of the AP and HC groups. Our finding demostrated that differences in the oral microbiome could distinguish AP from healthy individuals. This difference was mainly manifested in the overabundance of opportunistic pathogenic bacteria *Prevotella, Actinomyces, Peptostreptococcus, Selenomonas*, and *Atopobium*, and the reduction of beneficial bacteria *Streptococcus, Neisseria* and *Gemella*. The destruction of this oral microbiota might affect the severe course of AP by aggravating the inflammatory response and other ways. In the future, we will study the causal relationship between oral flora changes and acute pancreatitis and use it for clinical diagnosis and intervention to provide new ideas for treating severe AP patients. Oral microbiota-based biomarkers can be used as a non-invasive way to detect AP, but this needs to be confirmed with larger samples.

## Supplementary Material

Supplemental Material

## References

[1] Shah AU, Sarwar A, Orabi AI, et al. Protease activation during in vivo pancreatitis is dependent on calcineurin activation. Am J Physiol Gastrointest Liver Physiol. 2009;297(5):G967–13. doi: 10.1152/ajpgi.00181.2009 20501444 PMC2777459

[2] Banks PA, Bollen TL, Dervenis C, et al. Classification of acute pancreatitis-2012: revision of the Atlanta classification and definitions by international consensus. Gut. 2013;62(1):102–111. doi: 10.1136/gutjnl-2012-302779 23100216

[3] Vege SS, Gardner TB, Chari ST, et al. Low mortality and high morbidity in severe acute pancreatitis without organ failure: a case for revising the Atlanta classification to include “moderately severe acute pancreatitis”. Am J Gastroenterol. 2009;104(3):710–715. doi: 10.1038/ajg.2008.77 19262525

[4] Hagjer S, Kumar N. Evaluation of the BISAP scoring system in prognostication of acute pancreatitis - a prospective observational study. Int J Surg. 2018;54(Pt A):76–81. doi: 10.1016/j.ijsu.2018.04.026 29684670

[5] Lankisch PG, Warnecke B, Bruns D, et al. The APACHE II score is unreliable to diagnose necrotizing pancreatitis on admission to hospital. Pancreas. 2002;24(3):217–222. doi: 10.1097/00006676-200204000-00002 11893927

[6] Slocum C, Kramer C, Genco CA. Immune dysregulation mediated by the oral microbiome: potential link to chronic inflammation and atherosclerosis. J Intern Med. 2016;280(1):114–128. doi: 10.1111/joim.12476 26791914

[7] Willis JR, Gabaldón T. The human oral microbiome in health and disease: from sequences to ecosystems. Microorganisms. 2020;8(2). doi: 10.3390/microorganisms8020308 PMC7074908 32102216

[8] Michaud DS, Izard J, Wilhelm-Benartzi CS, et al. Plasma antibodies to oral bacteria and risk of pancreatic cancer in a large European prospective cohort study. Gut. 2013;62(12):1764–1770. doi: 10.1136/gutjnl-2012-303006 22990306 PMC3815505

[9] Fan X, Alekseyenko AV, Wu J, et al. Human oral microbiome and prospective risk for pancreatic cancer: a population-based nested case-control study. Gut. 2018;67(1):120–127. doi: 10.1136/gutjnl-2016-312580 27742762 PMC5607064

[10] Kamada N, Chen GY, Inohara N, et al. Control of pathogens and pathobionts by the gut microbiota. Nat Immunol. 2013;14(7):685–690. doi: 10.1038/ni.2608 23778796 PMC4083503

[11] Pushalkar S, Hundeyin M, Daley D, et al. The pancreatic cancer microbiome promotes oncogenesis by induction of innate and adaptive immune suppression. Cancer Discovery. 2018;8(4):403–416. doi: 10.1158/2159-8290.Cd-17-1134 29567829 PMC6225783

[12] Del Castillo E, Meier R, Chung M, et al. The microbiomes of pancreatic and duodenum tissue overlap and are highly subject specific but differ between pancreatic cancer and noncancer subjects. Cancer epidemiology, biomarkers & prevention. 2019;28(2):370–383. doi: 10.1158/1055-9965.Epi-18-0542 PMC6363867 30373903

[13] Brook I, Frazier EH. Microbiological analysis of pancreatic abscess. Clin Infect Dis. 1996;22(2):384–385. doi: 10.1093/clinids/22.2.384 8838210

[14] E DG, S LR, X ZJ, et al. Microbiota restricts trafficking of bacteria to mesenteric lymph nodes by CX(3)CR1(hi) cells. Nature. 2013;494(7435):116–120. doi: 10.1038/nature11809 23334413 PMC3711636

[15] Beger HG, Bittner R, Block S, et al. Bacterial contamination of pancreatic necrosis. A prospective clinical study. Gastroenterology. 1986;91(2):433–438. doi: 10.1016/0016-5085(86)90579-2 3522342

[16] Binder Gallimidi A, Fischman S, Revach B, et al. Periodontal pathogens Porphyromonas gingivalis and Fusobacterium nucleatum promote tumor progression in an oral-specific chemical carcinogenesis model. Oncotarget. 2015;6(26):22613–22623. doi: 10.18632/oncotarget.4209 26158901 PMC4673186

[17] Costello EK, Lauber CL, Hamady M, et al. Bacterial community variation in human body habitats across space and time. Science. 2009;326(5960):1694–1697. doi: 10.1126/science.1177486 19892944 PMC3602444

[18] UK guidelines for the management of acute pancreatitis. Gut. 2005;54(Suppl 3):iii1–9. doi: 10.1136/gut.2004.057026 15831893 PMC1867800

[19] Khumaedi AI, Purnamasari D, Wijaya IP, et al. The relationship of diabetes, periodontitis and cardiovascular disease. Diabetes Metab Syndr. 2019;13(2):1675–1678. doi: 10.1016/j.dsx.2019.03.023 31336540

[20] Zhang X, Zhang D, Jia H, et al. The oral and gut microbiomes are perturbed in rheumatoid arthritis and partly normalized after treatment. Nature Med. 2015;21(8):895–905. doi: 10.1038/nm.3914 26214836

[21] Read E, Curtis MA, Neves JF. The role of oral bacteria in inflammatory bowel disease. Nat Rev Gastroenterol Hepatol. 2021;18(10):731–742. doi: 10.1038/s41575-021-00488-4 34400822

[22] Lapidot Y, Amir A, Ben-Simon S, et al. Alterations of the salivary and fecal microbiome in patients with primary sclerosing cholangitis. Hepatol Int. 2021;15(1):191–201. doi: 10.1007/s12072-020-10089-z 32949377

[23] Zhang C, Hu A, Li J, et al. Combined non-invasive prediction and New biomarkers of oral and fecal microbiota in patients with gastric and colorectal cancer. Front Cell Infect Microbiol. 2022;12:830684. doi: 10.3389/fcimb.2022.830684 35663463 PMC9161364

[24] Wei AL, Li M, Li GQ, et al. Oral microbiome and pancreatic cancer. World J Gastroenterol. 2020;26(48):7679–7692. doi: 10.3748/wjg.v26.i48.7679 33505144 PMC7789059

[25] Lu H, Ren Z, Li A, et al. Tongue coating microbiome data distinguish patients with pancreatic head cancer from healthy controls. J Oral Microbiol. 2019;11(1):1563409. doi: 10.1080/20002297.2018.1563409 30728915 PMC6352935

[26] Zhu Y, He C, Li X, et al. Gut microbiota dysbiosis worsens the severity of acute pancreatitis in patients and mice. J Gastroenterol. 2019;54(4):347–358. doi: 10.1007/s00535-018-1529-0 30519748

[27] F LH, Li A, Zhang T, et al. Disordered oropharyngeal microbial communities in H7N9 patients with or without secondary bacterial lung infection. Emerg Microbes Infect. 2017;6(12):e112. doi: 10.1038/emi.2017.101 29259328 PMC5750457

[28] Ren Z, Li A, Jiang J, et al. Gut microbiome analysis as a tool towards targeted non-invasive biomarkers for early hepatocellular carcinoma. Gut. 2019;68(6):1014–1023. doi: 10.1136/gutjnl-2017-315084 30045880 PMC6580753

[29] Feng Q, Liang S, Jia H, et al. Gut microbiome development along the colorectal adenoma–carcinoma sequence. Nat Commun. 2015;6(1):6528. doi: 10.1038/ncomms7528 25758642

[30] Farrell JJ, Zhang L, Zhou H, et al. Variations of oral microbiota are associated with pancreatic diseases including pancreatic cancer. Gut. 2012;61(4):582–588. doi: 10.1136/gutjnl-2011-300784 21994333 PMC3705763

[31] Couvigny B, de Wouters T, Kaci G, et al. Commensal Streptococcus salivarius modulates PPARγ transcriptional activity in human intestinal epithelial cells. PLoS One. 2015;10(5):e0125371. doi: 10.1371/journal.pone.0125371 25946041 PMC4422599

[32] Zupancic K, Kriksic V, Kovacevic I, et al. Influence of oral probiotic Streptococcus salivarius K12 on ear and oral cavity health in humans: systematic review. Probiotics and antimicrobial proteins 2017;9(2):102–110. 28236205 10.1007/s12602-017-9261-2

[33] Cosseau C, A DD, Dullaghan E, et al. The commensal Streptococcus salivarius K12 downregulates the innate immune responses of human epithelial cells and promotes host-microbe homeostasis. Infect Immun. 2008;76(9):4163–4175. doi: 10.1128/iai.00188-08 18625732 PMC2519405

[34] Sun H, Zhao X, Zhou Y, et al. Characterization of oral microbiome and exploration of potential biomarkers in patients with pancreatic cancer. Bio Med Res Int. 2020;2020:1–11. doi: 10.1155/2020/4712498 PMC7652608 33204698

[35] Ríos-Covián D, Ruas-Madiedo P, Margolles A, et al. Intestinal short chain fatty acids and their link with diet and human health. Front Microbiol. 2016;7:185. doi: 10.3389/fmicb.2016.00185 26925050 PMC4756104

[36] Kelly CJ, Zheng L, Campbell EL, et al. Crosstalk between microbiota-derived short-chain fatty acids and intestinal epithelial HIF augments tissue barrier function. Cell Host Microbe. 2015;17(5):662–671. doi: 10.1016/j.chom.2015.03.005 25865369 PMC4433427

[37] Said HS, Suda W, Nakagome S, et al. Dysbiosis of salivary microbiota in inflammatory bowel disease and its association with oral immunological biomarkers. DNA Res. 2014;21(1):15–25. doi: 10.1093/dnares/dst037 24013298 PMC3925391

[38] Ortiz AP, Acosta-Pagán KT, Oramas-Sepúlveda C, et al. Oral microbiota and periodontitis severity among Hispanic adults. Front Cell Infect Microbiol. 2022;12:965159. doi: 10.3389/fcimb.2022.965159 36452304 PMC9703052

[39] Chattopadhyay I, Verma M, Panda M. Role of oral microbiome signatures in diagnosis and prognosis of oral cancer . Technology in cancer research & treatment. 2019;18:1533033819867354. doi: 10.1177/1533033819867354 PMC6676258 31370775

[40] Mager DL, Haffajee AD, Devlin PM, et al. The salivary microbiota as a diagnostic indicator of oral cancer: a descriptive, non-randomized study of cancer-free and oral squamous cell carcinoma subjects. J Transl Med. 2005;3(1):27. doi: 10.1186/1479-5876-3-27 15987522 PMC1226180

[41] Kondo Y, Sato K, Nagano K, et al. Involvement of PorK, a component of the type IX secretion system, in Prevotella melaninogenica pathogenicity. Microbiol Immunol. 2018;62(9):554–566. doi: 10.1111/1348-0421.12638 30028034

[42] Han M, Liu G, Chen Y, et al. Comparative genomics uncovers the genetic diversity and characters of Veillonella atypica and provides insights into its potential applications. Front Microbiol. 2020;11:1219. doi: 10.3389/fmicb.2020.01219 32655519 PMC7324755

[43] Lv L, Jiang H, Chen X, et al. The salivary microbiota of patients with primary biliary cholangitis is distinctive and pathogenic. Front Immunol. 2021;12:713647. doi: 10.3389/fimmu.2021.713647 34367180 PMC8335641

[44] Yang SF, Huang HD, Fan WL, et al. Compositional and functional variations of oral microbiota associated with the mutational changes in oral cancer. Oral Oncol. 2018;77:1–8. doi: 10.1016/j.oraloncology.2017.12.005 29362114

[45] Xun Z, Zhang Q, Xu T, et al. Dysbiosis and ecotypes of the salivary microbiome associated with inflammatory bowel diseases and the assistance in diagnosis of diseases using oral bacterial profiles. Front Microbiol. 2018;9:1136. doi: 10.3389/fmicb.2018.01136 29899737 PMC5988890

[46] Kwun JS, Kang SH, Lee HJ, et al. Comparison of thrombus, gut, and oral microbiomes in Korean patients with ST-elevation myocardial infarction: a case-control study. Exp Mol Med. 2020;52(12):2069–2079. doi: 10.1038/s12276-020-00543-1 33339953 PMC8080616

[47] Li Y, Cui J, Liu Y, et al. Tongue-Coating microbiota, and metabolic disorders: a novel area of interactive research. Front Cardiovasc Med. 2021;8:730203. doi: 10.3389/fcvm.2021.730203 34490384 PMC8417575

[48] R KA, Pushalkar S, Gulivindala D, et al. Periodontal dysbiosis associates with reduced CSF Aβ42 in cognitively normal elderly. Alzheimer’s & Dementia (Amsterdam, Netherlands). 2021;13(1):e12172. doi: 10.1002/dad2.12172 PMC8040436 33869725

[49] Rastogi D, Ratner AJ, Prince A. Host-bacterial interactions in the initiation of inflammation. Paediatr Respir Rev. 2001;2(3):245–252. doi: 10.1053/prrv.2001.0147 12052326

[50] Colin R, Ni B, Laganenka L, et al. Multiple functions of flagellar motility and chemotaxis in bacterial physiology. FEMS Microbiol Rev. 2021;45(6). doi: 10.1093/femsre/fuab038 PMC8632791 34227665

[51] Ribet D, Cossart P. How bacterial pathogens colonize their hosts and invade deeper tissues. Microbes Infect. 2015;17(3):173–183. doi: 10.1016/j.micinf.2015.01.004 25637951

[52] Li Q, Wang C, Tang C, et al. Bacteremia in patients with acute pancreatitis as revealed by 16S ribosomal RNA gene-based techniques*. Crit Care Med. 2013;41(8):1938–1950. doi: 10.1097/CCM.0b013e31828a3dba 23863226

[53] Yang N, Zhang DL, Hao JY. Coagulopathy and the prognostic potential of D-dimer in hyperlipidemia-induced acute pancreatitis. Hepatobiliary Pancreatic Dis Int. 2015;14(6):633–641. doi: 10.1016/s1499-3872(15)60376-9 26663012

[54] Yatheendranathan GD, GiriPrasanna A, Thirunavukarasu VS. Letter to the editor concerning the publication: “evaluation of the BISAP scoring system in prognostication of acute pancreatitis - a prospective observational study”. Int J Surg. 2019;65:86–87. doi: 10.1016/j.ijsu.2019.03.013 30910683

[55] Sublette MG, Cross TL, Korcarz CE, et al. Effects of smoking and smoking cessation on the intestinal microbiota. J Clin Med. 2020;9(9):2963. doi: 10.3390/jcm9092963 32937839 PMC7564179

