# Supplementary material for: Alterations of oral microbiota are associated with the development and severity of acute pancreatitis
Source: J Oral Microbiol. 2023 Oct 5;15(1):2264619. doi: 10.1080/20002297.2023.2264619 (PMC10557549; doi:10.1080/20002297.2023.2264619)
Supplement: Supplemental Material [file ZJOM_A_2264619_SM5844.zip › Supplementary files/Figure S2.pdf]

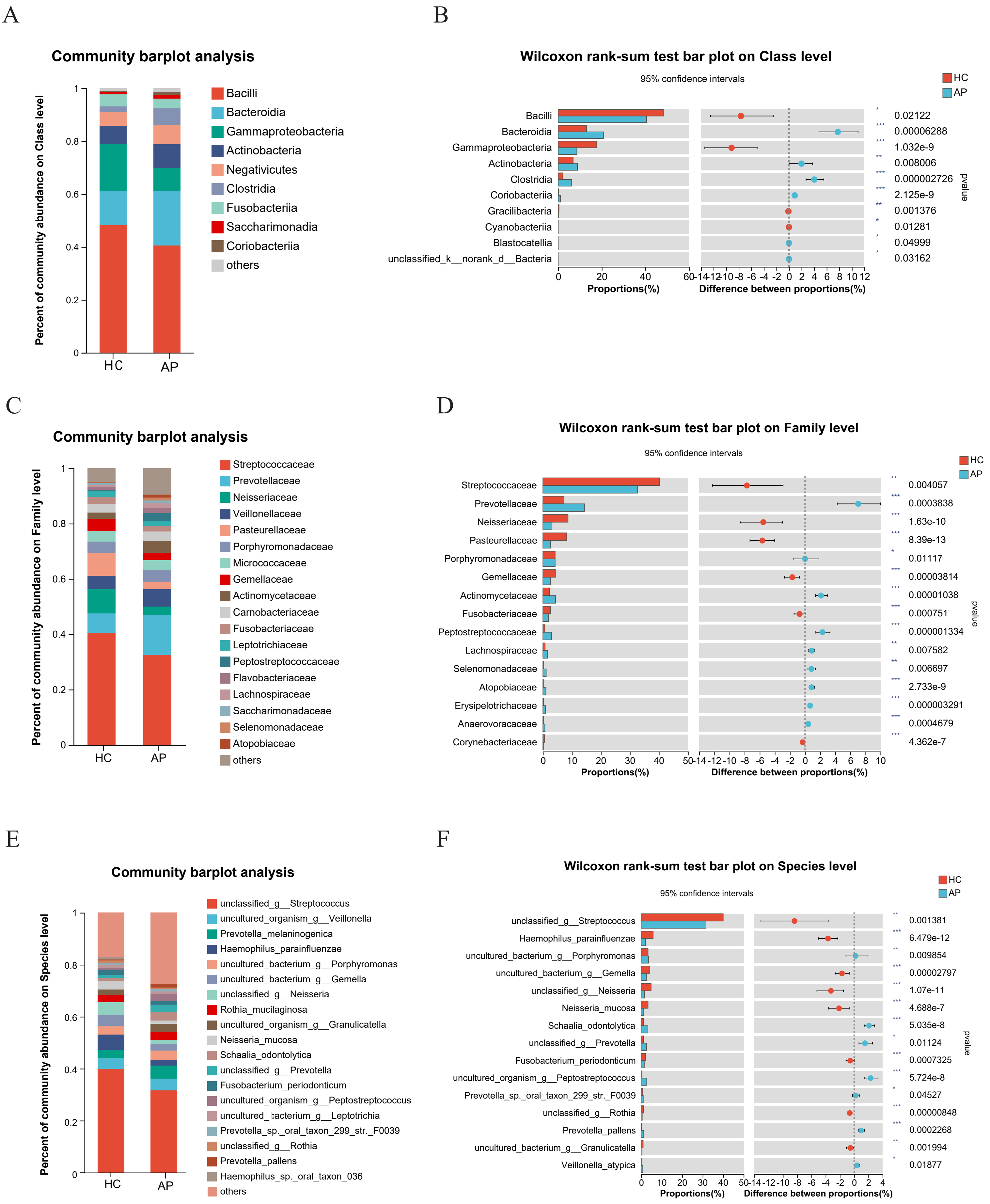

Figure S2.The different composition of class,family and species of HC and AP group.
(A)Histogram of microbial composition at the class level.(B)The significant different class between two groups.
(C)Histogram of microbial composition at the family level.(D)The significant different family between two groups.
(E)Histogram of microbial composition at the species level.(F)The significant different species between two groups.
