# Supplementary material for: Alterations of oral microbiota are associated with the development and severity of acute pancreatitis
Source: J Oral Microbiol. 2023 Oct 5;15(1):2264619. doi: 10.1080/20002297.2023.2264619 (PMC10557549; doi:10.1080/20002297.2023.2264619)
Supplement: Supplemental Material [file ZJOM_A_2264619_SM5844.zip › Supplementary files/Figure S3.pdf]

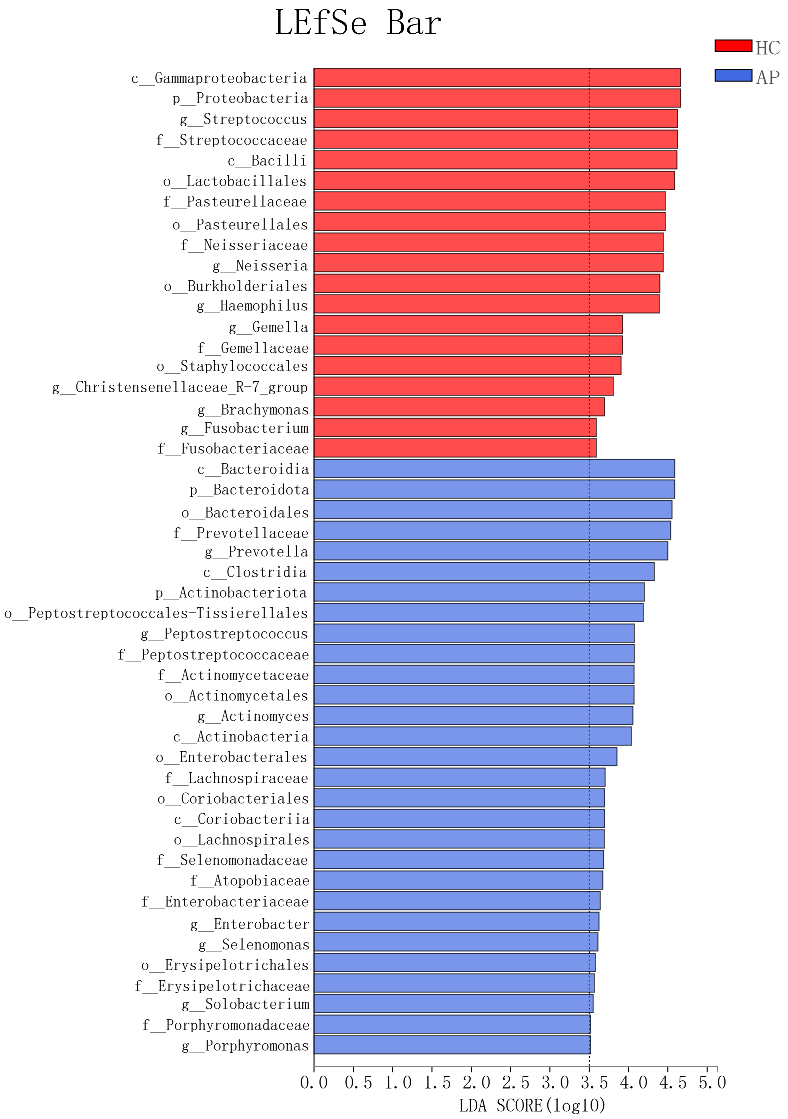

Figure S3.Microbes of differential abundance were plotted as a histogram with an LDA score＞3.5. LDA score histogram showed the oral microbiome with significant differences between the two groups.
