# Supplementary material for: Alterations of oral microbiota are associated with the development and severity of acute pancreatitis
Source: J Oral Microbiol. 2023 Oct 5;15(1):2264619. doi: 10.1080/20002297.2023.2264619 (PMC10557549; doi:10.1080/20002297.2023.2264619)
Supplement: Supplemental Material [file ZJOM_A_2264619_SM5844.zip › Supplementary files/Figure S4.pdf]

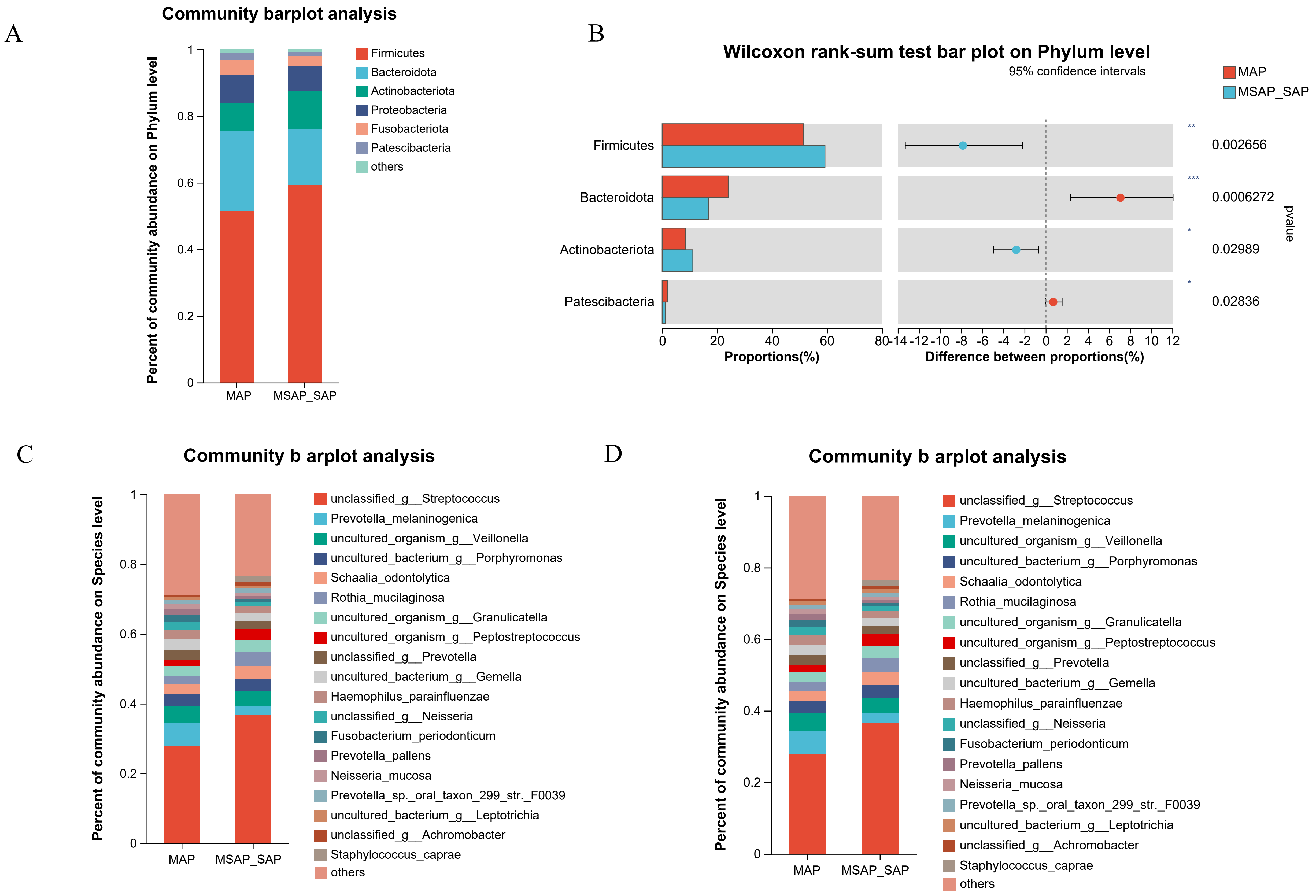

Figure S4.The different composition of phylum,and species of MAP and MSAP_SAP group. (A)Histogram of microbial composition at the phylum level.(B)The significant different phylum between two groups. (C)Histogram of microbial composition at the species level.(D)The significant different species between two groups.
